# Duration of SARS-CoV-2 shedding: A population-based, Canadian study

**DOI:** 10.1371/journal.pone.0252217

**Published:** 2021-06-17

**Authors:** Susan P. Phillips, Xuejiao Wei, Jeffrey C. Kwong, Jonathan Gubbay, Kevin L. Schwartz, Anna Majury, Patti A. Groome

**Affiliations:** 1 Department of Family Medicine, Queen’s University, Kingston, Ontario, Canada; 2 Public Health Sciences, Queen’s University, Kingston, Ontario, Canada; 3 ICES Queen’s, Queen’s University, Kingston, ON, Canada; 4 ICES, Toronto, Ontario, Canada; 5 Dalla Lana School of Public Health, University of Toronto, Toronto, Ontario, Canada; 6 Department of Pediatrics University of Toronto, Toronto, Ontario, Canada; 7 Faculty of Health Sciences, Queen’s University, Kingston, Ontario, Canada; German Centre for Neurodegenerative Diseases Site Munich: Deutsches Zentrum fur Neurodegenerative Erkrankungen Standort Munchen, GERMANY

## Abstract

**Introduction:**

There is an evidence gap regarding the duration of SARS-CoV-2 shedding and of its variability across different care settings and by age, sex, income, and co-morbidities. Such evidence is part of understanding of infectivity and reinfection. We examine direct measures of viral shedding using a linked population-based health administrative dataset.

**Methods:**

Laboratory and sociodemographic databases for Ontario, Canada were linked to identify those testing positive (RT-PCR) between Jan. 15 and April 30, 2020 who underwent subsequent testing by May 31, 2020. To maximise use of available data, we computed two shedding duration estimates defined as the time between initial positive and most recent positive (documented shedding) or second of two negative tests (documented resolution). We also report multivariable results using quantile regression to examine subgroup differences.

**Results:**

In Ontario, of the 16,595 who tested positive before April 30, 2020, 6604 had sufficient subsequent testing to allow shedding duration calculation. Documented shedding median duration calculated in 4,889 (29% of 16,595) patients was 19 days (IQR 12–28). Documented resolution median duration calculated in 3,219 (19% of the 16,595) patients was 25 days (IQR 18–34). Long-term care residents had 3–5 day longer shedding durations using both definitions. Shorter documented shedding durations of 2–4 days were observed in those living in higher income neighbourhoods. Shorter documented resolution durations of 2–3 days were observed at the 25^th^% of the distribution in those aged 20–49. Only 11.5% of those with definitive negative test results reverted to negative status by day 14.

**Conclusions:**

Viral shedding continued well beyond 14 days among this large subset of a population-based group with COVID-19, and longer still for long-term care residents and those living in less affluent neighborhoods. Our findings do not speak to duration of infectivity but are useful for understanding the expected duration of RT-PCR positivity and for identifying reinfection.

## Background

With modelling from similar respiratory viruses as the only available guide [1], SARS-CoV-2 infectivity was initially assumed to resolve within 14 days of symptom onset. Subsequent research on molecular detection using real-time reverse transcriptase PCR (RT-PCR) suggested that SARS-CoV-2 shedding begins 2 to 3 days prior to symptom onset and continues for 7 to14 days [2–9], but for up to 20 to 31 days with more severe infection [4, 5, 10–12]. This evidence lacked external validity as almost all studies had been conducted in single settings, and were limited to hospitalised patients. Reliability was also difficult to determine as most lacked peer review [3, 6–8, 10], specimen sources varied [1–4, 8], and sample sizes were small. More recently, among a somewhat larger sample (n = 150) of the <10% of test positive individuals from one US academic centre who were retested, median time from positive to negative RT-PCR was 17.5 days [13]. In a different US study, the median duration of viral shedding as measured by time from a positive PCR to the second of two negative tests among 668 patients was 20.9 days [14]. That study tracked those with repeat positive or negative tests and computed a documented shedding estimate based on continuing positivity of 15 days. None of the studies referenced above were population-based and, with two exceptions [14, 15] they excluded censored cases (those that do not have a shedding resolution time).

Robust, population-level evidence regarding the duration of viral shedding as measured by RT-PCR, whether it varies with age, sex, socioeconomic status, illness severity, or co-morbidities is lacking. Such information could be a useful step in understanding the link between viral shedding and infectivity [16, 17] and the utility of PCR testing for re-infection. This study describes the duration of RT-PCR-tested SARS-CoV-2 detection, henceforth referred to as viral shedding, using laboratory reports from the entire population of Ontario, Canada among individuals who had follow-up testing after an initial positive finding. We also assess variability in the duration of shedding by known determinants of health in general, such as age, sex, socioeconomic status, residence in long-term care (LTC), and selected chronic conditions.

## Methods

### Population

Our study included individuals with a first positive RT-PCR test performed in Ontario prior to April 30, 2020 who then had at least one subsequent positive test and/or two consecutive negative tests by May 31, 2020. Throughout the study period Ontario’s policy was to retest test-positive healthcare workers and hospitalised patients by 14 days after symptom onset and until they had two consecutive negative tests (NN) [18].

### Data

The Ontario Laboratories Information System (OLIS) database tracks 80 to 85% of all RT-PCR testing for SARS-CoV-2. OLIS includes information on specimen source (e.g. nasopharyngeal) and can be linked to ICES-held individual-level demographic and health data holdings. ICES is an independent, non-profit research institute whose legal status under Ontario’s health information privacy law allows it to collect and analyze health care and demographic data, without consent, for health system evaluation and improvement. Data holdings used for this study were the Registered Persons Database (RPDB) for age, sex and vital status, the Canadian Institute for Health Information (CIHI) Discharge Abstract Database for hospitalisation and intensive care admissions, the National Ambulatory Care Reporting System for emergency department (ED) admissions, and to identify those residing in LTC, the Ontario Health Information Plan physician billing claims database, the Ontario Drug Benefit database and the Continuing Care Reporting System. Postal codes of home addresses linked to census data were used to assign area-level socioeconomic status. Only patients with specimens taken from the upper or lower respiratory tract were included. All datasets were linked using unique encoded identifiers and were analysed at ICES.

### Study variables

Date of onset of viral shedding was assigned as the day of a first positive RT-PCR test as this was available and more definitive than self-reported date of symptom onset, particularly for those with no symptoms. Duration was calculated using the same approach taken by Agarwal et al [14]. “Documented shedding” was defined as the time to last positive test in those with at least one follow-up positive test. “Documented resolution” was defined as the time to the second negative test after the last positive test in those with two such consecutive negative tests. Our use of the documented shedding estimate reduced selection bias by allowing inclusion of those patients whose testing ceased (generally because of death, symptom resolution, or the end date of the study) without definitive negative findings. Study follow-up was truncated on May 31, 2020. LTC residence was assigned to anyone living in LTC in the 90 days prior to date of onset. Ambulatory care patients were identified as non-LTC residents with no hospital admission during the study period. Hospitalised patients were anyone hospitalised for any reason during their follow-up time. Chronic conditions (asthma, congestive heart failure, chronic obstructive lung disease, hypertension and diabetes) were identified via established prevalent disease cohorts at ICES created using validated algorithms applied to health administrative data. Socioeconomic status was assessed using area-level income quintiles at the census dissemination level (geographic units of 400 to 700 persons). Deaths occurring any time between a first positive test and May 31, 2020, were tracked for subset reporting.

### Statistical analysis

Overall study population characteristics are described. We also compared those characteristics by whether or not patients contributed to the documented resolution and documented shedding estimates. Statistical comparisons were made using standardised differences [19], z-tests and chi-square tests. We report the median, 25^th^, and 75^th^ percentiles with 95% confidence intervals of the documented resolution and documented shedding durations for all patients and for the subsets: LTC residents, hospitalised patients, ICU patients, those presenting in emergency (ED) at any time during follow up, and ambulatory patients (the remainder). We also report results for those who died versus the rest and for those diagnosed up to March 31, 2020 to assess whether longer follow up would yield longer estimates. To document the amount of information we had available, we report the number and percent positive tests performed for each follow up day in an S1 Appendix. We assessed differences in time to documented resolution and documented shedding by age, sex, socioeconomic status, LTC residency, hospitalisation status and selected chronic conditions using multivariable quantile regressions computed at the 25^th^, median, and 75^th^ percentiles.

### Ethics

The study received ethics approval from the Queen’s University Faculty of Health Sciences Research Ethics Board. As only anonymised administrative data were used the REB waived any requirement for consents.

### Results

From the first available positive test on February 22, 2020, until April 30, 2020, there were 16,595 Ontario residents who tested positive for SARS-CoV-2 on either a nasal or throat swab. Excluded from this study were the 8,332 who were not retested, 1,659 who had insufficient testing to assign one of our two duration estimates (most because there was only one negative result after the first positive) or were non-residents of Ontario. This resulted in a study population of 6,604 (39.8% of all Ontarians testing positive), who underwent a total of 24,816 follow-up tests (mean 3.8 per person, SD 1.7) over a median follow-up time of 26 days (IQR 17–35, range 1–83). The number of tests per day and the percent positive on each day is reported in the S1 Appendix for all study participants and separately for those contributing to the documented shedding and documented resolution durations. Repeat testing was performed on between 150 and 914 patients for each follow-up day, with the largest numbers of repeat tests occurring on days 13 (578 tests) through 22 (603 tests). Daily positive test rates varied from 33% to 93% within 30 days of the first positive test.

Table 1 describes the overall study population (n = 6,604). Their average age was 57.9 years (SD 21.2), 64.5% were female, hypertension was the most common chronic condition (45.7%), and those in lower income quintile areas were over-represented. Noting all relevant groups for each patient, which is why percentages do not total 100, 20.8% were living in LTC, 20.9% were hospitalised, 6.8% were in the ICU, 31.8% were seen in the ED and 63.5% were deemed to be in the ambulatory group. Overall, 355 (5.4%) died during the follow-up period.

**Table 1:** Baseline characteristics at time of first positive test for study cohort overall and for subgroups contributing to documented shedding and documented resolution estimates (n (%) unless otherwise stated).

	Overall Study Population	Documented Shedding Group	Remainder *(those with only 2 negative tests post-positive)*	SD	P-value	Documented Resolution Group	Remainder *(those without 2 negative tests post-positive)*	SD	P-value
N	6,604	4,889	1,715			3,219	3,385		
Age—mean (St. Dev.)	57.9 (21.2)	58.6 (21.7)	56.0 (19.8)	0.12	< .0001	56.0 (19.9)	59.7 (22.3)	0.18	< .0001
Age—median (Q1-Q3)	56 (42–76)	57 (42–78)	54 (41–69)	0.12	< .0001	54 (41–70)	59 (42–80)	0.18	< .0001
Age Groups									
0–19	68 (1.0%)	57 (1.2%)	11 (0.6%)	0.06	< .0001	21 (0.7%)	47 (1.4%)	0.07	< .0001
20–29	595 (9.0%)	462 (9.4%)	133 (7.8%)	0.06		278 (8.6%)	317 (9.4%)	0.03	
30–39	802 (12.1%)	566 (11.6%)	236 (13.8%)	0.07		415 (12.9%)	387 (11.4%)	0.04	
40–49	998 (15.1%)	683 (14.0%)	315 (18.4%)	0.12		583 (18.1%)	415 (12.3%)	0.16	
50–59	1,219 (18.5%)	861 (17.6%)	358 (20.9%)	0.08		681 (21.2%)	538 (15.9%)	0.14	
60–69	906 (13.7%)	669 (13.7%)	237 (13.8%)	0		430 (13.4%)	476 (14.1%)	0.02	
70–79	594 (9.0%)	462 (9.4%)	132 (7.7%)	0.06		264 (8.2%)	330 (9.7%)	0.05	
80+	1,422 (21.5%)	1,129 (23.1%)	293 (17.1%)	0.15		547 (17.0%)	875 (25.8%)	0.22	
Sex—female	4,257 (64.5%)	3,069 (62.8%)	1,188 (69.3%)	0.14	< .0001	2,197 (68.3%)	2,060 (60.9%)	0.16	< .0001
Chronic Diseases									
Asthma	1,061 (16.1%)	815 (16.7%)	246 (14.3%)	0.06	0.02	496 (15.4%)	565 (16.7%)	0.03	0.16
CHF	672 (10.2%)	521 (10.7%)	151 (8.8%)	0.06	0.03	299 (9.3%)	373 (11.0%)	0.06	0.02
COPD	838 (12.7%)	647 (13.2%)	191 (11.1%)	0.06	0.02	369 (11.5%)	469 (13.9%)	0.07	0.004
HBP	3,015 (45.7%)	2,279 (46.6%)	736 (42.9%)	0.07	0.008	1,388 (43.1%)	1,627 (48.1%)	0.1	< .0001
Diabetes	1,702 (25.8%)	1,304 (26.7%)	398 (23.2%)	0.08	0.005	775 (24.1%)	927 (27.4%)	0.08	0.002
Neighbourhood income									
1 (Lowest quintile)	1,789 (27.1%)	1,276 (26.1%)	513 (29.9%)	0.08	0.004	912 (28.3%)	877 (25.9%)	0.05	0.06
2	1,452 (22.0%)	1,061 (21.7%)	391 (22.8%)	0.03		719 (22.3%)	733 (21.7%)	0.02	
3	1,293 (19.6%)	989 (20.2%)	304 (17.7%)	0.06		609 (18.9%)	684 (20.2%)	0.03	
4	1,031 (15.6%)	774 (15.8%)	257 (15.0%)	0.02		502 (15.6%)	529 (15.6%)	0	
5 (Highest quintile)	994 (15.1%)	760 (15.5%)	234 (13.6%)	0.05		452 (14.0%)	542 (16.0%)	0.06	
Unknown	45 (0.7%)	29 (0.6%)	16 (0.9%)	0.04		25 (0.8%)	20 (0.6%)	0.02	
LTC Resident	1,371 (20.8%)	1,092 (22.3%)	279 (16.3%)	0.15	<0.001	515 (16.0%)	856 (25.3%)	0.23	<0.0001
Hospitalised	1,377 (20.9%)	1,125 (23.0%)	252 (14.7%)	0.21	< .0001	656 (20.4%)	721 (21.3%)	0.02	0.36
ICU	447 (6.8%)	371 (7.6%)	76 (4.4%)	0.13	< .0001	226 (7.0%)	221 (6.5%)	0.02	0.43
ED visit	2,100 (31.8%)	1,621 (33.2%)	479 (27.9%)	0.11	< .0001	998 (31.0%)	1,102 (32.6%)	0.03	0.18
Ambulatory Group (non-LTC, non-hospital/ICU)	4,194 (63.5%)	2,957 (60.5%)	1,237 (72.1%)	0.25	< .0001	2,207 (68.6%)	1,987 (58.7%)	0.21	< .0001
Died during follow-up	355 (5.4%)	319 (6.5%)	36 (2.1%)	0.22	< .0001	52 (1.6%)	303 (9.0%)	0.33	< .0001

SD: Standardized Difference; CHF: Congestive Heart Failure; COPD: Chronic Obstructive Pulmonary Disease; HBP: Hypertension; LTC: Long-Term Care; ICU: Intensive Care Unit; ED: Emergency Department.

Table 1 also presents descriptions of those contributing to each of the shedding duration estimates, comparing those subgroups to the remainder of the study population. Of the 6,604 patients in this study, 1,504 (22.8%) contributed to both duration estimates, 3,385 (51.3%) contributed to the documented shedding estimate only and 1,715 (26.0%) contributed to the documented resolution estimate only. Focussing on standardised differences above 0.10, the group contributing to the documented shedding calculation (i.e., those who had at least one positive repeat test) were older at 58.6 years than the remainder (who consisted of those with only negative PCRs after their initial positive) at 56.0 years. The documented shedding group were also more likely to be male than the remainder (37.2% versus 30.7%), more likely to reside in LTC (22.3% versus 16.3%), more likely to be hospitalised (23.0% versus 14.7%) more likely to be admitted to ICU (7.6% versus 4.4%) and to have used the ED (33.2% versus 27.9%) and therefore less likely to be deemed ‘ambulatory’ (60.5% versus 72.1%). More of those documented shedding patients died during the study period compared to the remainder (6.5% versus 2.1%). The group contributing to the documented resolution estimate (i.e., those with two negative PCRs after a positive) were younger at 56.0 years than the remainder (those without 2 negative tests post positive) at 59.7 years. The documented resolution group were also less likely to be male than the remainder (31.7% versus 39.1%), less likely to have hypertension (43.1% versus 48.1%), less likely to be in LTC (16.0% versus 25.3%) and more likely to be ambulatory (68.6% versus 58.7%). Fewer of these documented resolution patients died during the study period compared to the remainder (1.6% versus 9.0%).

**Fig 1:**
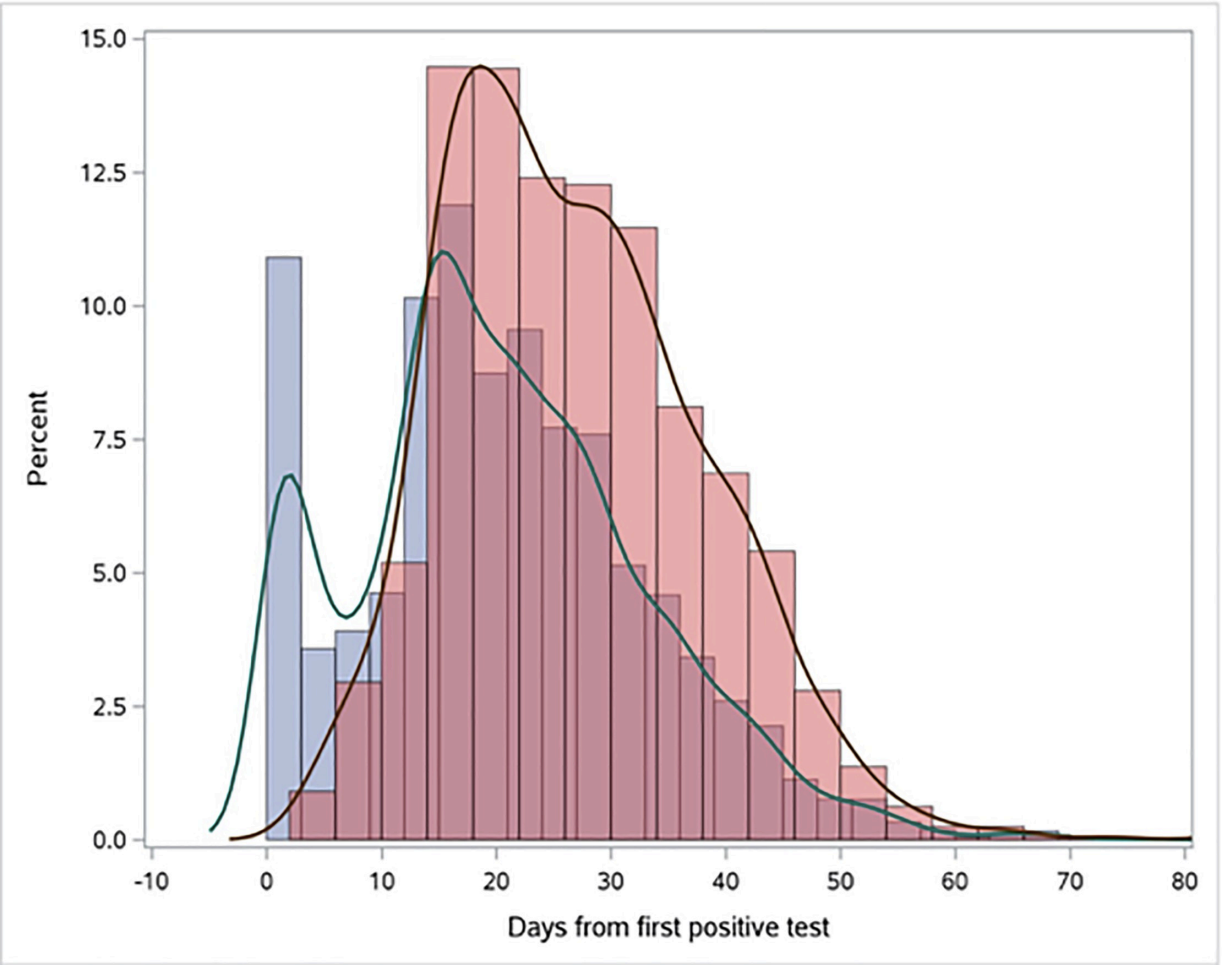
Histograms and smoothed density plots of the documented shedding (blue) and documented resolution (red) distributions.

Fig 1 presents smoothed density plots showing shedding duration distributions for those with two negative follow-up COVID tests (documented resolution) and those without such resolution (documented shedding). Overall, the documented shedding median shedding duration was 19 days (95% CI 19–20) with an IQR of 12–28 days and 33.2% (95% CI, 31.8%-34.5%) had resolved by the 14-day mark. The documented resolution median shedding duration was 25 days (95% CI 25–26) with an IQR of 18–34 days and 11.5% (95% CI, 10.4%-12.7%) had resolved by 14 days. Patients living in LTC had longer durations as did hospitalised patients, and those in ICU.

Table 2 reports summary statistics for the documented shedding and documented resolution durations by selected subgroups. Compared to the whole study population, LTC residents had longer documented shedding and documented resolution durations (medians of 23 and 29 days compared to 19 and 25), and fewer individuals with documented shedding durations of less than 15 days (23.1% versus 33.2%). Patients who were hospitalised, were in the ICU or were seen in the ED had longer documented resolution durations (medians of 29, 30 and 28 days respectively versus 25 days) and fewer individuals who were in the ICU or ED had durations of less than 15 days (2.7% in ICU and 7.1% in ED). Patients who died during the study period had shorter documented shedding durations (median of 10 days) and 63.3% had documented shedding durations of less than 15 days. This group had little impact on the overall results as the estimates in those who did not die are very similar to the overall results. The 60-day follow-up subset (those diagnosed before April 1, 2020) had documented resolution results that were 2–4 days longer than the overall study population. Their documented shedding duration was shorter at the 25^th^ percentile by 7 days and the median by 3 days. Consequently, more of those patients had a shedding duration of less than 14 days (45.3%) compared to the overall population (33.2%).

**Table 2:** Shedding duration distribution summary descriptions for the documented shedding (no test resolution) and documented resolution (two negative PCR tests after a positive) duration definitions, overall study population and by subgroup.

Group	Duration	N	Mean (SD)	25% (95% CI)	Median (95% CI)	75% (95% CI)	% Duration <15 days (95% CI)
Overall	Documented shedding	4889	20.2 (12.5)	12 (12–13)	19 (19–20)	28 (27–28)	33.2% (31.8%-34.5%)
	Documented resolution	3219	26.6 (11.2)	18 (17–18)	25 (25–26)	34 (33–35)	11.5% (10.4%-12.7%)
Long Term Care	Documented shedding	1092	23.6 (12.5)	15 (14–16)	23 (22–24)	31 (30–33)	23.1% (20.6%-25.7%)
	Documented resolution	515	29.7 (11.5)	21 (19–23)	29 (28–31)	39 (36–40)	9.3% (7.0%-12.2%)
Hospitalised	Documented shedding	1125	20.8 (14.1)	8 (8–10)	20 (19–22)	30 (29–31)	38.1% (35.3%-41.0%)
	Documented resolution	656	29.1 (11.5)	20 (19–22)	29 (28–30)	37 (35–38)	8.2% (6.2%-10.6%)
ICU	Documented shedding	371	20.1 (13.4)	10 (8–12)	20 (18–22)	28 (27–31)	37.7% (32.8%-42.9%)
	Documented resolution	226	30.5 (10.6)	22 (21–24)	30 (28–31)	37 (35–39)	2.7% (1.0%-5.7%)
ED	Documented shedding	1621	20.5 (13.9)	10 (8–11)	19 (18–20)	29 (28–31)	37.1% (34.7%-39.5%)
	Documented resolution	998	29.0 (11.5)	20 (19–21)	28 (27–29)	37 (36–38)	7.1% (5.6%-8.9%)
Ambulatory	Documented shedding	2957	19.2 (11.7)	13 (12–13)	18 (17–18)	26 (26–27)	34.3% (32.6%-36.0%)
	Documented resolution	2207	25.4 (10.9)	17 (17–18)	23 (23–24)	32 (32–33)	12.7% (11.4%-14.2%)
Died during follow-up	Documented shedding	319	11.8 (9.8)	3 (2–3)	10 (7–12)	19 (17–21)	63.3% (57.8%-68.6%)
	Documented resolution	52	23.6 (11.4)	16 (8–20)	23 (19–29)	31 (28–37)	21.2% (11.1%-34.7%)
Did not die during follow-up	Documented shedding	4570	20.8 (12.4)	13 (13–14)	20 (19–20)	28 (28–29)	31.1% (29.7%-32.4%)
	Documented resolution	3167	26.7 (11.2)	18 (18–18)	25 (25–26)	34 (33–35)	11.4% (10.3%-12.5%)
60-day follow-up	Documented shedding	905	17.9 (14.7)	5 (4–7)	16 (15–17)	25 (24–27)	45.3% (42.0%-48.6%)
	Documented resolution	601	29.8 (13.4)	20 (18–21)	28 (27–29)	38 (36–40)	9.5% (7.3%-12.1%)

SD: Standard Deviation; CI: Confidence Interval; ICU: Intensive Care Unit; ED: Emergency Department

Tables 3 and 4 report on the multivariable quantile regression results at the 25^th^, 50^th^ (median), and 75^th^ percentiles for the documented shedding and documented resolution durations. LTC residents had longer durations for both, ranging from 3 to 5 days longer than non-LTC residents. Other findings vary significantly across the two duration estimates. For the documented shedding estimates (Table 3), persons living in the wealthiest neighbourhoods had shorter durations than the rest by 2 to 4 days. The median duration was 2 days longer in women and those with congestive heart failure and 2 days shorter in 20- to 29-year olds. The 25^th^ percentile was 2 days longer in women and 3 days shorter in 70- to 79-year olds and those who were hospitalised, indicating narrower and wider left tails, respectively for this population’s shedding duration distribution. Patients over age 80 had a right skewed distribution with the 75^th^ percentile being 3 days longer than the reference. The documented resolution estimates (Table 4) indicate 2–3 day longer intervals in hospitalised patients. The 25^th^ percentile was 2–3 days shorter in patients aged 20–49 compared to those aged 50–59 (wider left tail) and the median was 2 days shorter in the 30-to 39-year age group.

**Table 3:** Multivariable quantile regression, factors associated with shedding duration (N = 4889).

	Shedding duration	25th percentile difference in days	Median difference in days	75th percentile difference in days
Characteristic	Median (IQR)	Estimate (95% CI)	Estimate (95% CI)	Estimate (95% CI)
Age				
0–19	18 (12–28)	-1.00 (-7.70, 5.70)	-0.25 (-5.08, 4.58)	1.00 (-4.02, 6.02)
20–29	16 (12–25)	-0.80 (-2.09, 0.49)	-2.00 (-3.47, -0.53)	-1.50 (-3.47, 0.47)
30–39	17 (13–26)	-0.00 (-1.33, 1.33)	-0.75 (-2.41, 0.91)	-0.50 (-2.47, 1.47)
40–49	18 (13–26)	0.20 (-0.90, 1.30)	-0.25 (-1.65, 1.15)	-0.50 (-2.36, 1.36)
50–59	19 (12–27)	Ref	Ref	Ref
60–69	19 (10–29)	-1.80 (-3.88, 0.28)	-0.50 (-2.17, 1.17)	1.50 (-0.28, 3.28)
70–79	21 (9–29)	-2.80 (-5.12, -0.48)	-1.00 (-3.06, 1.06)	1.50 (-0.87, 3.87)
80+	22 (14–31)	-0.40 (-2.19, 1.39)	-0.25 (-2.03, 1.53)	3.00 (0.81, 5.19)
Sex—female	20 (13–28)	1.80 (0.99, 2.61)	1.75 (0.90, 2.60)	1.00 (-0.15, 2.15)
SES				
1(low)	19 (13–28)	3.60 (2.30, 4.90)	2.75 (1.45, 4.05)	2.50 (0.62, 4.38)
2	20 (14–28)	3.80 (2.54, 5.06)	2.75 (1.34, 4.16)	2.50 (0.89, 4.11)
3	20 (13–28)	3.00 (1.59, 4.41)	3.75 (2.27, 5.23)	2.50 (0.69, 4.31)
4	19 (12–28)	2.80 (1.34, 4.26)	2.50 (1.01, 3.99)	3.00 (0.91, 5.09)
5	17 (9–27)	Ref	Ref	Ref
unknown	24 (14–33)	4.80 (-0.50, 10.10)	5.25 (-2.12, 12.62)	6.50 (0.40, 12.60)
Chronic Diseases				
Asthma	19 (12–30)	-0.40 (-1.69, 0.89)	0.25 (-1.05, 1.55)	2.00 (0.41, 3.59)
CHF	23 (14–33)	0.60 (-1.28, 2.48)	2.25 (0.52, 3.98)	2.00 (-0.24, 4.24)
COPD	21 (12–30)	-1.20 (-3.01, 0.61)	0.25 (-1.33, 1.83)	-0.50 (-2.20, 1.20)
HBP	21 (12–30)	-0.00 (-0.96, 0.96)	0.25 (-0.86, 1.36)	0.00 (-1.46, 1.46)
Diabetes	21 (12–30)	-0.60 (-1.78, 0.58)	0.00 (-1.10, 1.10)	-0.00 (-1.34, 1.34)
LTC	23 (15–31)	4.00 (2.43, 5.57)	4.75 (3.52, 5.98)	2.00 (0.31, 3.69)
Hospitalised	20 (8–30)	-3.20 (-4.75, -1.65)	0.75 (-0.57, 2.07)	2.00 (0.50, 3.50)

IQR: Inter-quartile range; CI: Confidence Interval; SES: Socioeconomic status measured by area-level median income quintiles; CHF: Congestive Heart Failure; COPD: Chronic Obstructive Pulmonary Disease; HBP: Hypertension; LTC: Long Term Care residents.

**Table 4:** Multivariable quantile regression results on factors associated with documented resolution duration (N = 3,219).

	Shedding duration	25th percentile difference in days	Median difference in days	75th percentile difference in days
Characteristic	Median (IQR)	Estimate (95% CI)	Estimate (95% CI)	Estimate (95% CI)
Age				
0–19	26 (16–35)	-3.00 (-10.5, 4.52)	2.00 (-8.42, 12.42)	3.50 (-3.57, 10.57)
20–29	23 (16–33)	-3.00 (-4.43, -1.57)	-2.00 (-4.75, 0.75)	0.58 (-1.63, 2.80)
30–39	23 (16–31)	-3.00 (-4.30, -1.70)	-2.00 (-3.49, -0.51)	-1.67 (-3.80, 0.47)
40–49	24 (17–32)	-2.00 (-3.13, -0.87)	-1.00 (-2.47, 0.47)	-0.25 (-2.10, 1.60)
50–59	26 (19–33)	Ref	Ref	Ref
60–69	26 (19–36)	-1.00 (-2.58, 0.58)	-1.00 (-3.05, 1.05)	1.50 (-0.78, 3.78)
70–79	28 (18–37)	-2.00 (-4.30, 0.30)	-2.00 (-4.76, 0.76)	1.25 (-1.58, 4.08)
80+	28 (20–37)	-1.00 (-3.03, 1.03)	-1.00 (-3.43, 1.43)	1.50 (-1.03, 4.03)
Sex—female	25 (18–34)	0.00 (-0.80, 0.80)	0.00 (-1.01, 1.01)	-0.25 (-1.51, 1.01)
SES				
1(low)	25 (18–34)	0.00 (-1.30, 1.30)	1.00 (-0.63, 2.63)	-0.50 (-2.29, 1.29)
2	25 (18–33)	0.00 (-1.38, 1.38)	1.00 (-0.71, 2.71)	-0.50 (-2.31, 1.31)
3	26 (18–35)	-0.00 (-1.59, 1.59)	2.00 (-0.02, 4.02)	0.83 (-1.14, 2.80)
4	26 (18–35)	0.00 (-1.39, 1.39)	2.00 (0.12, 3.88)	1.58 (-0.66, 3.83)
5	24 (18–34)	Ref	Ref	Ref
unknown	29 (19–33)	2.00 (-5.79, 9.79)	6.00 (-0.60, 12.60)	-0.50 (-10.4, 9.38)
Chronic Diseases				
Asthma	26 (18–36)	1.00 (-0.10, 2.10)	1.00 (-0.37, 2.37)	1.08 (-0.56, 2.73)
CHF	28 (18–39)	-1.00 (-3.12, 1.12)	0.00 (-2.32, 2.32)	2.08 (-0.28, 4.45)
COPD	27 (19–36)	-1.00 (-2.77, 0.77)	-1.00 (-2.71, 0.71)	-0.50 (-2.47, 1.47)
HBP	27 (19–36)	0.00 (-0.92, 0.92)	1.00 (-0.42, 2.42)	0.75 (-0.85, 2.35)
Diabetes	27 (19–36)	-1.00 (-2.15, 0.15)	0.00 (-1.36, 1.36)	0.00 (-1.64, 1.64)
LTC	29 (21–39)	3.00 (1.11, 4.89)	5.00 (3.20, 6.80)	3.00 (1.09, 4.91)
Hospitalised	29 (20–37)	3.00 (1.50, 4.50)	3.00 (1.70, 4.30)	2.17 (0.56, 3.77)

IQR: Inter-quartile range; CI: Confidence Interval; SES: Socioeconomic status measured by area-level median income quintiles; CHF: Congestive Heart Failure; COPD: Chronic Obstructive Pulmonary Disease; HBP: Hypertension; LTC: Long Term Care residents.

## Discussion

Although ongoing positive RT-PCR testing now appears unlikely to indicate continuing infectivity as was thought early in the pandemic, it remains the most reliable and available test for diagnosing SARS-CoV-2. To the best of our knowledge and at the time of writing, ours was the first large study to explore the course of shedding of viral RNA among those with COVID-19. We provide population-level documentation of the duration and time to resolution of that shedding including whether it varies by age, sex, area-level income, co-morbidities, LTC residency, and hospitalisation status. Approximately 40% of all those infected across Ontario during the study period were retested because until the end of May 2020 the RT-PCR test was assumed to be a proxy indicator of ongoing infectious risk. It was also used as a ’test of cure’ for healthcare workers and hospitalised patients [18].

Overall, 67% of those with ongoing PCR+ testing (documented shedding) and 88% of patients whose PCR testing reverted to negative indicating resolution (documented resolution) continued to shed measurable viral RNA more than 14 days after a first positive RT-PCR. The median documented shedding duration was 19 days and the median documented resolution was 25 days. Medians of these two durations for hospitalised patients were 20 and 29 days, respectively. In keeping with findings from much of the earlier, smaller, hospital-based research viral shedding continued longer than was initially assumed [4, 5, 9, 11–15]. As prior evidence did not examine duration of viral shedding specifically among LTC residents their medians of 23 and 29 days have no comparators. Both documented shedding and documented resolution were statistically significantly longer in a multivariable regression for those in LTC and the documented resolution was longer amongst hospitalised patients, adding 2 to 3 days to their shedding duration compared to the rest.

Patients contributing to our documented shedding estimate (i.e. those with ongoing positive PCR tests) were older than the remainder of our study population and more likely to be living in LTC (Table 1). The duration of testing for a disproportionate number within this group was limited by death. This could have shortened the apparent duration measured by the documented shedding and led to its underestimation. In this documented shedding group, and in contrast to results by Xiao *et al*, women had 2 days longer shedding durations at the 25^th^ percentile and the median compared with men in the same group [15]. Whether this is because of sex/gender variations in immune responses, confounding by other characteristics like age or location (LTC), or because of the female disproportion of healthcare providers who all underwent continued testing to determine when they could return to work cannot be determined. Those over age 80 or with congestive heart failure also had more protracted shedding durations (3 days longer at the 75^th^ percentile and 2 days longer at the median, respectively). Documented shedding duration, while constant across income quintiles 1 (lowest) to 4, was 3 to 4 days shorter for residents of Ontario’s wealthiest neighbourhoods. This socioeconomic gradient, not examined by others, may reflect more ready access to repeat testing among wealthier patients, or a lower viral ’load’ at onset. There are data suggesting that the initial viral load, determined to some extent by ability to distance from potential carriers of infection, has an impact on severity and duration of COVID-19 [20].

Patients contributing to the documented resolution estimate (i.e. who had 2 consecutive follow-up negative tests) were younger, more likely to be female, less likely to be in LTC, and were much more likely to have survived than the remainder group (Table 1). Many among them would have been healthcare workers. Young people (20–49 years old) had a 2- to 3-day shorter documented resolution duration at the 25^th^ percentile of the distribution compared to the 50–59-year-old reference group. Those age 30–49 had a shorter documented resolution duration of 2 days at the median compared to that reference. This may reflect more rapid resolution of infection among younger populations, a longer duration between exposure to infection and initial testing, or their seeking earlier retesting and determination of negative PCR status that would allow for a return to work. 1.6% of those in the documented resolution group died within our short study timeframe. Whether these deaths were unrelated to the infection, itself, or, more probably, evidence that the end of viral shedding does not indicate resolution of illness arising from the virus cannot be determined.

A consistent and novel finding from these data is that those in LTC had a longer duration of viral shedding. Explanations for this might include their older age and disproportion of women and co-morbidities, or possible exposure to a greater viral load because of limited isolation options [20]. We cannot rule out lead-time bias; in some LTC facilities when outbreaks occurred all contacts were tested, resulting in an unknown number of positive individuals identified prior to symptom onset.

In contrast to many prior studies [2–10, 13] which examined data only from hospitalised patients with test-based proof of resolution, we included non-hospitalised patients and those whose follow-up testing remained positive. This strategy allowed us to uncover subgroup differences that we otherwise would not have observed. Our more inclusive strategy also decreased the potential for selection bias. Our study population consisted of 39.8% of all of the province’s test-positive individuals and it included all of the 19.4% who had documented resolution durations in OLIS by May 30, 2020. Compared to reports describing all Ontarians testing positive as of April 30, 2020, our study population included more women (57.4% versus 64.5%). The age distribution of the study population was, however, very similar to that of the whole Ontario test-positive population, with the largest difference in the 40- to 59-year old group (30.1% in the overall test positive group and 33.6% in our study) [21]. Over-representation of women among those tested and testing positive in Ontario and in our study subset is unlikely to arise from a sex-based predisposition to infection [22], but more probably reflects female occupational roles as health care workers who were disproportionately exposed and required to have repeat testing until negative.

### Strengths and limitations

Strengths of our study are the large, population-based sample, our ability to look at shedding patterns beyond those in hospitalised patients by including LTC residents and ambulatory individuals, the accuracy of test results, minimising of false negative reporting by only including those with a confirmatory second negative follow-up test in the documented resolution calculation, and some ability to assess the impact of deaths on estimates. Our research took advantage of a short timeframe (February through May 2020) during which repeat testing until resolution was required for hospitalised patients and healthcare providers. That situation allowed us to calculate documented resolution durations which are no longer estimable in such a (relatively) unselected population. We also provide a more complete picture of the SARS-CoV-2 shedding duration than most others have done by calculating a documented shedding estimate which was not restricted to those with two consecutive negative tests (demonstrated evidence of COVID shedding cessation).

Limitations of our study include lack of symptom onset data and bias in our documented shedding and documented resolution estimates. We did not have access to dates of symptom onset so our estimates begin on the day of an individual’s first positive test. Among the 3,385 (51.3%) patients in our study population who did not have two consecutive negative tests (69% of the documented shedding estimate group, 0% of the documented resolution group), repeated testing likely occurred only with ongoing illness, resulting in a longer documented shedding duration than if testing had been done at predetermined intervals on all positive patients. Without universal and repeated RT-PCR screening, findings cannot reflect the prevalence or course of viral shedding of those who were not tested, who may have had few, if any, symptoms and, perhaps, a shorter course of shedding. Our potential follow-up of a minimum of 30 days, dictated by changing testing policies, may have been too short to fully describe the durations we wished to examine. We looked at the subset who had a minimum of 60 days of potential follow-up and they did have a 2–4 day longer time to documented resolution, indicating our documented resolution results may be slight underestimates. Cycle threshold values (Ct) for PCR tests may have added depth to our findings but were not available in the accessed datasets [23]. Ontario has not undertaken whole population testing and therefore we cannot comment on the burden of disease at a population level but can only analyse the demographics and shedding patterns of those who were tested. Finally, viral shedding prior to symptom development and prior to testing was not measurable using available data but would lengthen the measured duration of shedding of RNA beyond what we have described.

## Conclusion

This study demonstrates that viral remnants remain detectable well beyond the 14-day period hypothesised early in the pandemic. This duration varies with patients’ sociodemographic characteristics. Clarifying the temporal relationship of initial and subsequent positive RT-PCR testing with ongoing infectivity, infection severity, prolongation of symptoms or sequelae of infection should be explored with further research. Our study of the course of viral shedding as measured by RT-PCR documented overall median shedding and resolution durations of 19 and 25 days, respectively. As SARS-CoV-2 shedding appears to continue well beyond infectivity other methods for determining the duration of infection are necessary, however, the information we have described adds to understanding of this virus and could guide use of RT-PCR testing to determine re-infection.

## Supporting information

S1 Appendix Positive test rates over time after initial positive test (95% error bars). (PDF)
