# Supplementary material for: Duration of SARS-CoV-2 shedding: A population-based, Canadian study
Source: PLoS One. 2021 Jun 17;16(6):e0252217. doi: 10.1371/journal.pone.0252217 (PMC8211234; doi:10.1371/journal.pone.0252217)

S1 Appendix. Positive test rates over time after initial positive test (95% error bars)

A. Overall study population

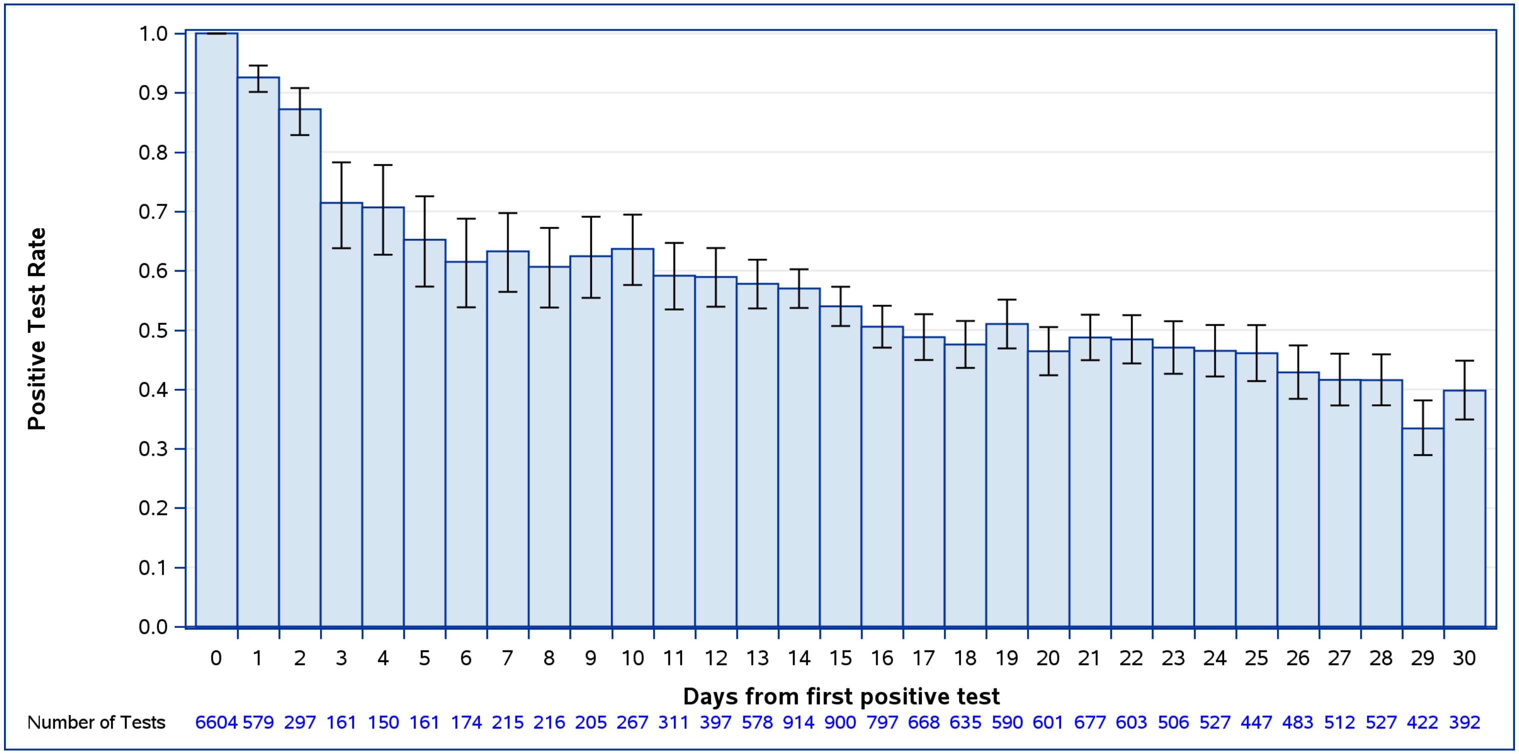

B. Documented shedding group

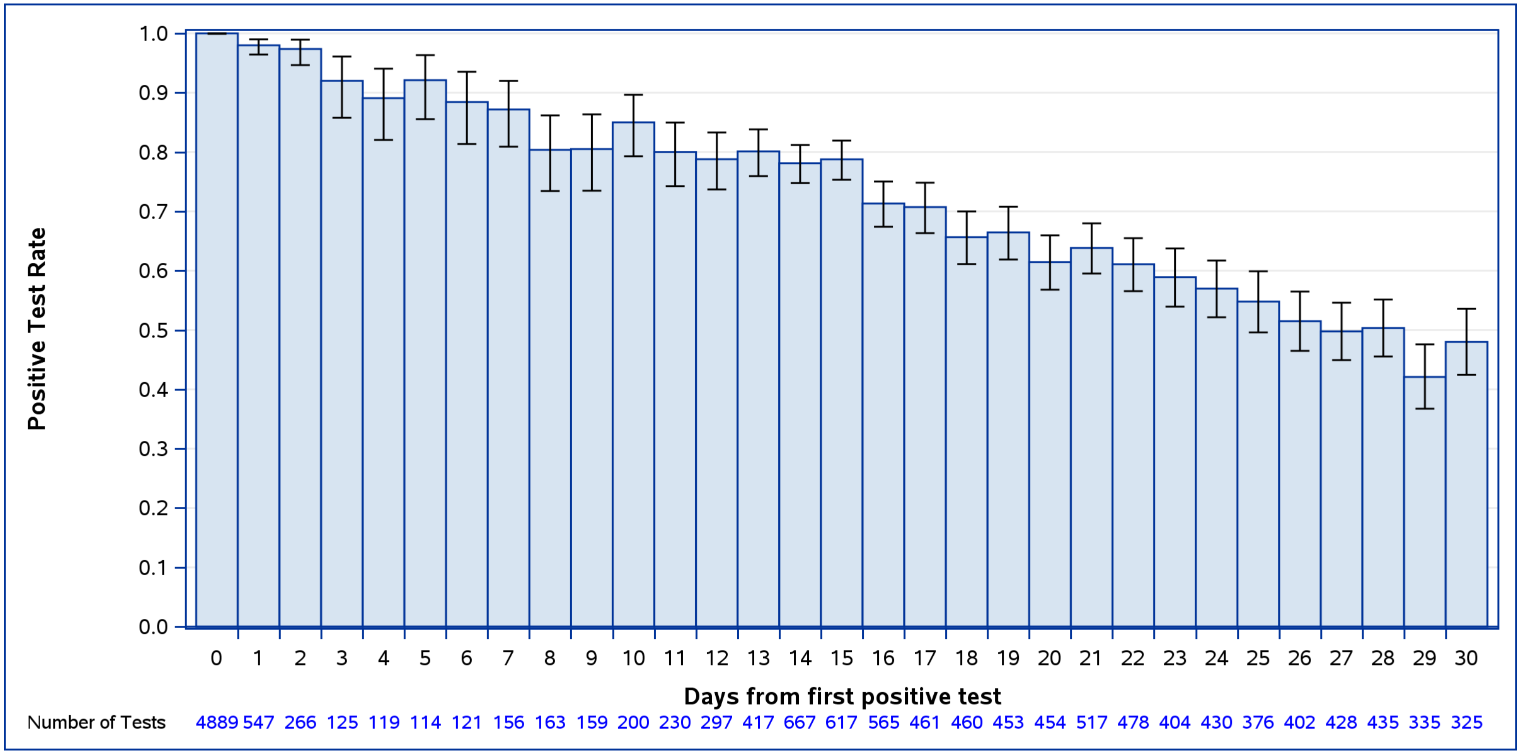

C. Documented resolution group

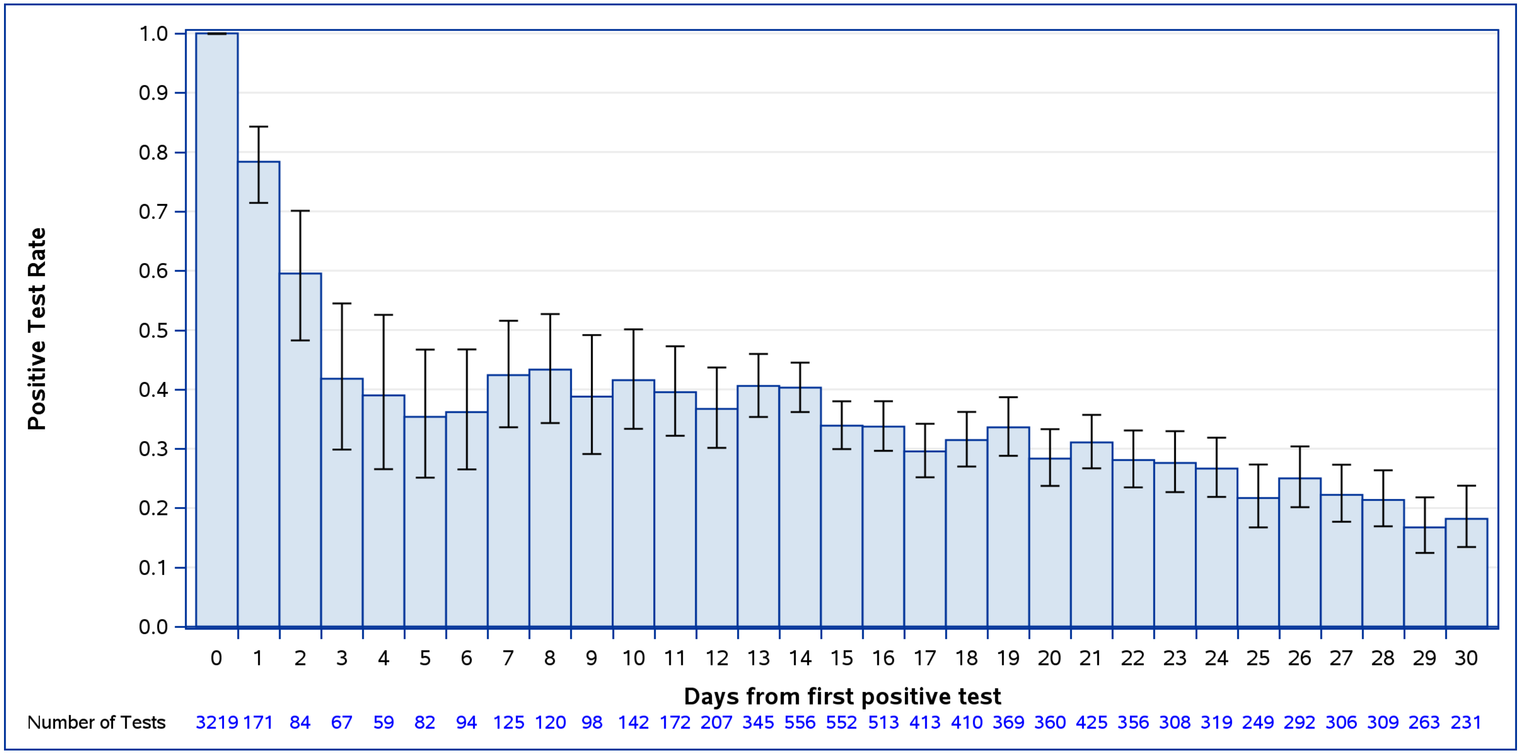

Supplement: S1 Appendix — (PDF) [file pone.0252217.s001.pdf]
